# Supplementary material for: Ultra-Stretchable Interconnects for High-Density Stretchable Electronics
Source: Micromachines (Basel). 2017 Sep 13;8(9):277. doi: 10.3390/mi8090277 (PMC6189761; doi:10.3390/mi8090277)
Supplement: Supplementary file 1 [file micromachines-08-00277-s001.zip › Supplementary Materials/Supplementary Materials.pdf]

Ultra-Stretchable Interconnects for High-Density Stretchable Electronics

Salman Shafqat, Johan P. M. Hoefnagels, Angel Savov, Shivani Joshi, Ronald Dekker and Marc G. D. Geers

1. Interconnect stretch required for circular detector array to stretch from flat to hemispherical state for particular fill factor:

A circular array with radius and thus initial length R, stretching to a hemisphere entails a final length of $(\pi/2) * R$ and thus a global strain of 57%. Using Equation (1) [1]:

$$Global\ device\ strain = \left(1 - \sqrt{fill\ factor}\,\right) * Global\ interconnect\ strain \quad (1)$$

For a fill factor of 0.9, with a global device strain of 57%, a global interconnect strain of 1110.7% is required.

2. Maximum von Mises stress in a guided cantilever beam with rectangular cross-sectional area:

For a beam with length l, and a rectangular cross-sectional area with width w and thickness t, being loaded as a guided cantilever beam on application of a point load W at the beam end, the end deflection δ_{max} is given by Equation (2) [2].

$$\delta_{max} = \frac{Wl^3}{12EI} \quad (2)$$

where E is the Young's Modulus and I is the area moment of inertia.

While the maximum stress σ_{max} in a beam in bending is given by the flexure formula:

$$\sigma_{max} = |M_{max}| * \frac{c}{I} \quad (3)$$

The maximum stress in this case is found at the top and bottom surface at the beam's ends, where c, i.e. the distance from the neutral axis to the top/bottom surfaces and is equal, i.e. $t/2$. Moreover, M_{max}, i.e. the maximum moment experienced by the beam, for this case, is given by Equation (3) [2].

$$M_{max} = \frac{Wl}{2} \quad (4)$$

Therefore,

$$\sigma_{max} = \frac{Wlt}{4I} \quad (5)$$

Inserting Equation (2) into Equation (5) yields:

$$\sigma_{max} = \frac{3\delta_{max}tE}{l^2} \quad (1)$$

For a guided cantilever beam with the boundaries free to contract in the transverse direction, the maximum von Mises $\sigma_{vm,max}$ stress is equal to the maximum stress σ_{max} in Equation (6). Note that the analysis above has been verified by performing an FE simulation of a guided cantilever beam with a rectangular cross-section.

3. Maximum von Mises stress in a torsion beam with rectangular cross-sectional area:

For a beam with a rectangular cross-sectional area, the maximum angle of twist θ_{max} on application of torque T is given by Equation (2) [3].

$$\theta_{max} = \frac{Tl}{k_2 b t^3 G} \quad (2)$$

where k_2 is a dimensionless coefficient that has been tabulated as a function of b/t. Some relevant values are listed in Table S1, where, l, b and t are the beam length, width and thickness respectively, while, G is the shear modulus.

The maximum shear stress τ_{max} in the beam is given by Equation (3) [3].

$$\tau_{max} = \frac{T}{k_1 b t^2} \quad (3)$$

where $k_1$is also a dimensionless coefficient listed in Table S1.

Inserting Equation (3) into Equation (2) provides:

$$\tau_{max} = \frac{k_2}{k_1}\frac{Gt}{l}\theta_{max} \quad (4)$$

where $k_1/k_1 = c$, which equals 1 for $b/t > 4$, and thus can be dropped from Equation (9).

Table S1. Dimensionless coefficients $k_1$and k_2 for a rectangular torsion bar [3].

b/t	1	2	3	4	6	8	∞
k_1	0.208	0.246	0.267	0.282	0.298	0.307	0.333
k_2	0.141	0.229	0.263	0.281	0.298	0.307	0.333

For a torsion beam with a rectangular cross-section with boundaries free to contract in the cross-sectional and longitudinal direction, the maximum von Mises stress $\sigma_{vm,max}$ is equal to $\sqrt{3}$ time the maximum shear stress τ_{max}, given by Equation (9). The analysis above has also been verified with an FE simulation of a torsion beam with rectangular cross-section.

4. Experimental results for parallel interconnects in plastic regime:

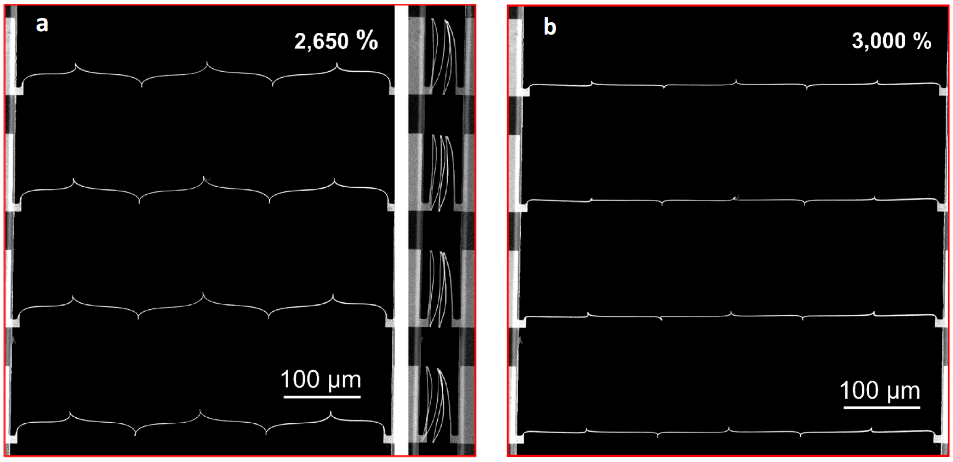

Figure S1. Experimental results of four parallel 100 µm-long interconnect structures stretched in the plastic regime. (**a**) Structures stretched beyond their elastic limit (left image) and subsequently unloaded (corresponding right image), which clearly shows accumulated plastic deformation. (**b**) Interconnect structures stretched to 3,000% global strain, close to the stretch to their full length. Even in the plastic regime, the four structures exhibit the same deformation behavior, demonstrating the reproducibility of the interconnect stretchability.

5. Accumulated equivalent plastic strain for increasing yield strength values:

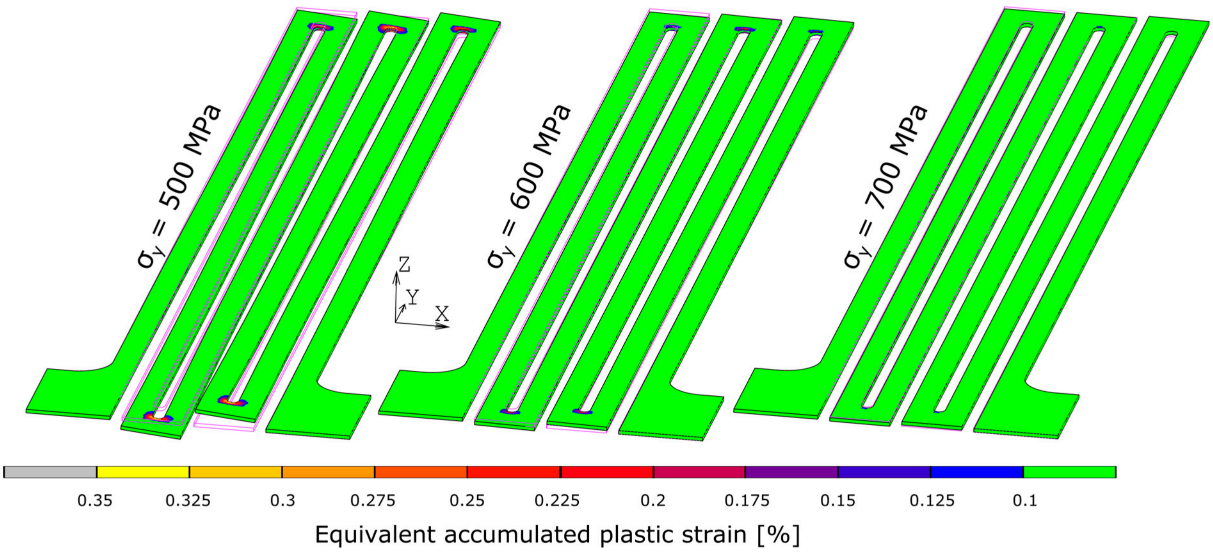

Figure S2. FE simulations showing the effect of accumulated plastic strains on the unloaded shape change of the interconnect for increasing values of σ_y used. Note that here 0.1% proof strain is used a definition of yielding. For $\sigma_y = 700$ MPa only negligible accumulated plastic strain and correspondingly in discernable shape change can be observed and thus is used as the predicted yield strength.

Reference

1. Zhang, Y.; Wang, S.; Li, X.; Fan, J.A.; Xu, S.; Song, Y.M.; Choi, K.J.; Yeo, W.H.; Lee, W.; Nazaar, S.N.; et al. Experimental and Theoretical Studies of Serpentine Microstructures Bonded To Prestrained Elastomers for Stretchable Electronics. *Adv. Funct. Mater.* **2014**, *24*, 2028–2037, doi:10.1002/adfm.201302957.
2. Young, W.C.; Budynas, R.G. *Roark's Formulas for Stress and Strain*; 2002; Vol. 7; ISBN 007072542X.
3. Gere, J.M.; Goodno, B.J. *Mechanics of materials*; Cengage Learning: Boston, MA, USA, 2009.

© 2017 by the authors; licensee MDPI, Basel, Switzerland. This article is an open access article distributed under the terms and conditions of the Creative Commons Attribution (CC BY) license (http://creativecommons.org/licenses/by/4.0/).
